# Synchronous mucosa-associated lymphoid tissue lymphoma in a married couple: a case report

**DOI:** 10.3389/fonc.2026.1870719

**Published:** 2026-06-29

**Authors:** Mo Cheng, Yafei Pi, Kexing Ren, Wenke Li, Liqun Zou

**Affiliations:** 1 Department of Head and Neck Oncology, Cancer Center, West China Hospital, Sichuan University, Chengdu, China; 2 Gastric Cancer Center, Division of Medical Oncology, West China Hospital, Sichuan University, Chengdu, China

**Keywords:** conjugal lymphoma, mucosa-associated lymphoid tissue lymphoma, common environmental exposure, synchronous occurrence, case report

## Abstract

Mucosa-associated lymphoid tissue (MALT) lymphoma represents an indolent form of non-Hodgkin lymphoma, predominantly arising in the gastrointestinal tract, particularly the stomach, with rare occurrences reported in the urinary tract. Its etiology is closely associated with chronic antigenic stimulation, commonly linked to persistent infections such as *H. pylori*, autoimmune conditions, or underlying genetic susceptibility. This report details a unique case of a married couple diagnosed with primary MALT lymphoma at separate anatomical sites—ileocecal region in the husband and urethra in the wife—within a span of one year. Both individuals attained complete remission following rituximab-based immunotherapy, supplemented by radiotherapy in wife case. The development of lymphoma in married couples, whether presenting with identical or distinct pathological subtypes, is exceptionally uncommon and frequently suggests potential shared environmental or infectious exposures.

## Introduction

Extranodal marginal zone lymphoma of mucosa-associated lymphoid tissue (MALT lymphoma) originates from the marginal zone B-cells of acquired MALT. It can arise in various anatomical sites, most commonly the gastrointestinal tract, and may also involve the ocular adnexa, thyroid, lungs, salivary glands, skin, among others (1). Primary involvement of the urethra or bladder is exceedingly rare. The pathogenesis is predominantly linked to chronic antigenic stimulation, such as persistent infection by *Helicobacter pylori* (*H. pylori*) in the stomach, *Borrelia burgdorferi* in cutaneous lesions, *Chlamydia psittaci* in ocular adnexal tissues, and *Campylobacter jejuni* in the small intestine, as well as to autoimmune diseases (2). Chronic inflammation and sustained immune activation create a microenvironment conducive to ongoing antigen-driven proliferation, genetic damage, the accumulation of molecular abnormalities, and eventual malignant transformation with clonal expansion. Furthermore, specific chromosomal translocations are associated with MALT lymphomagenesis, including t(14;18)(q32;q21), t(11;18)(q21;q21), t(1;14)(p22;q32), and t(3;14)(p14.1;q32). Among these, t(11;18)(q21;q21) is the most frequent and is clinically correlated with resistance to *H. pylori* eradication therapy and advanced-stage disease (3).

Herein, we report the unique case of a married couple who were both diagnosed with MALT lymphoma. The husband was treated with rituximab monotherapy, while the wife underwent transurethral resection followed by combined rituximab and radiotherapy. Both patients achieved complete remission. This conjugal presentation raises intriguing questions regarding potential shared environmental or infectious exposures contributing to lymphomagenesis in non−consanguineous individuals.

## Case description

### Case 1: the wife

**Figure 1:**
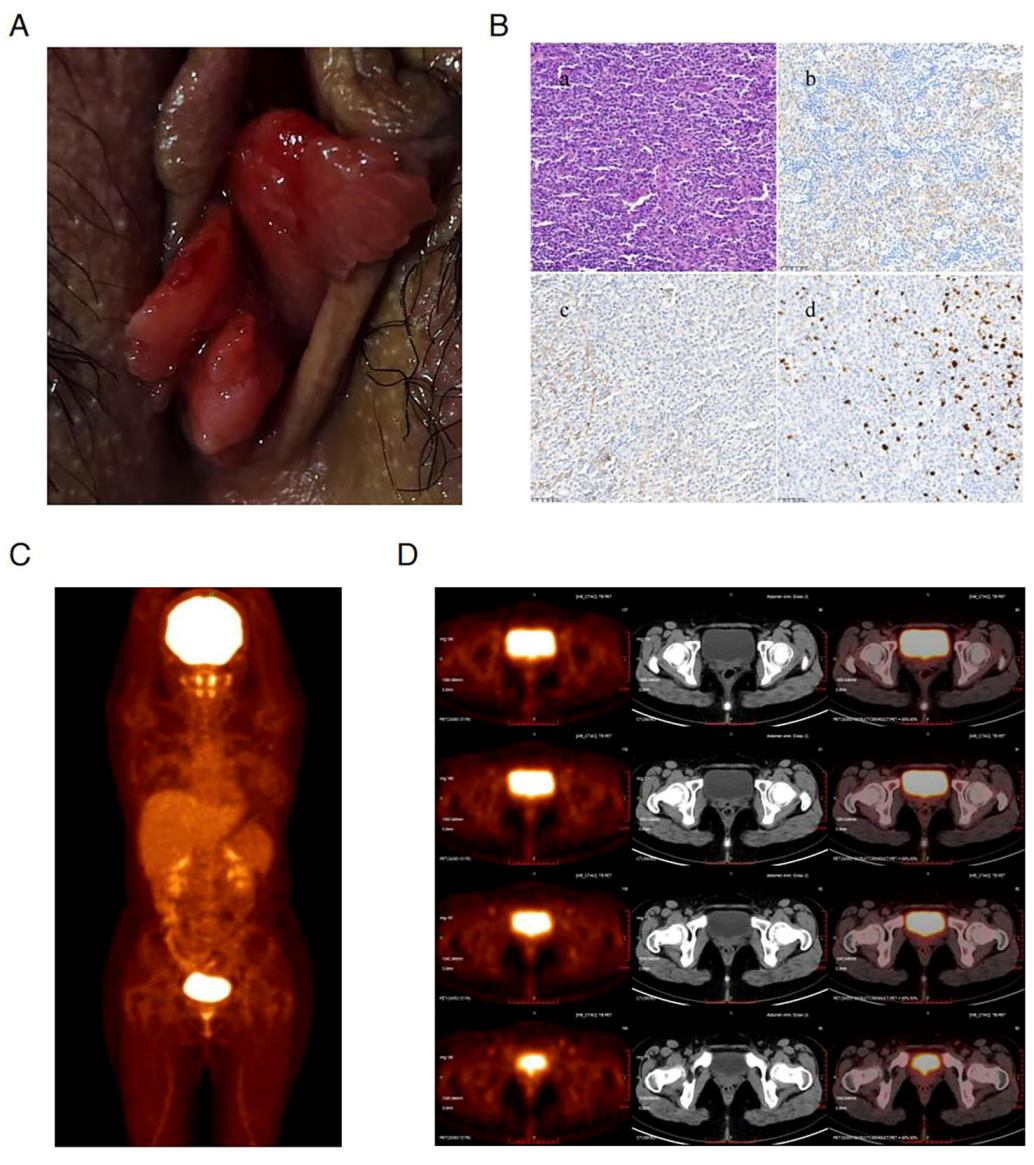
Pathological and imaging features of the wife’s urethral MALT lymphoma. **(A)** Tumor at initial presentation. **(B)** Histopathological and immunohistochemical characterization (original magnification: ×20). **(a)** Hematoxylin and eosin (H&E) staining. **(b)** CD20 shows scattered positive signals (positive, scattered). **(c)** CD3 is negative (negative). **(d)** Ki-67 proliferation index is low (positive, 3%–5%). **(C, D)** Postoperative PET/CT images reveal no evidence of residual tumor.

A 27-year-old primigravida at 28 weeks of gestation presented with a 4-month history of painless terminal gross hematuria and a painless mass at the urethral meatus. Physical examination revealed a firm, non-ulcerated mass measuring approximately 3 cm in diameter at the urethral orifice (**Figure 1A**). She reported associated urinary frequency and urgency but denied dysuria, fever, night sweats, or weight loss. There was no history of chronic urinary tract infections or other significant medical or family history.

Given the lesion’s indolent behavior (no growth over 4 months) and her advanced gestational age, a shared decision was made with the patient and her family to postpone surgical intervention until after delivery. Two months later, following an uncomplicated delivery, an abdominopelvic computed tomography (CT) scan was performed. It demonstrated a patchily enhancing lesion at the external urethral orifice measuring 37 × 40 mm, with a minimal amount of pelvic fluid. No regional lymphadenopathy was identified. All routine laboratory parameters were within normal limits. A 13C-urea breath test was performed and was negative. Serological tests for syphilis and human immunodeficiency virus were negative, and urine culture yielded no bacterial growth. Subsequently, the patient underwent transurethral resection of the lesion, excision of the urethral mass, and diagnostic flexible cystoscopy. Histopathological examination of the resected specimen confirmed the diagnosis of MALT lymphoma with plasmacytic differentiation (**Figure 1B**). Immunohistochemical staining was positive for CD20, CD79a, CD19, CD138 (majority), BCL2 (60–70%), BCL6 (weak/focal), and MUM-1 (majority); and negative for CD3, CD5, CD23, CD10, Cyclin D1, CD43, IgG4, and IgD. Staining for CD21 and CD23 revealed no follicular dendritic cell networks. The Ki-67 proliferation index was low (3–5%). *In situ* hybridization for Epstein-Barr virus-encoded RNA (EBER1/2) was negative. Polymerase chain reaction (PCR) with GeneScan analysis confirmed clonal rearrangements of the immunoglobulin heavy chain (IGH) and kappa light chain (IGK) genes. Postoperative restaging with ^18^F-fluorodeoxyglucose positron emission tomography/computed tomography (^18^F-FDG PET/CT) showed no definite abnormal bladder wall thickening or pathological FDG uptake in cervical, thoracic, or abdominal lymph nodes, though interpretation was partially limited by physiological urinary tracer activity (**Figures 1C,D**). A bone marrow biopsy revealed no lymphomatous involvement. Collectively, these findings indicated no evidence of residual or disseminated disease following surgery. The patient received 4 weekly infusions of rituximab (600 mg per dose), followed by consolidative local radiotherapy (12 fractions to a total dose of 20 Gy). Treatment was well-tolerated without significant adverse events. A follow-up pelvic magnetic resonance imaging (MRI) scan performed after completion of therapy showed no signs of local recurrence. The patient remains asymptomatic and disease-free at her last follow-up visit 9 months post-treatment.

### Case 2: the husband

The husband, a 32-year-old man, presented one year prior to his wife’s diagnosis with recurrent abdominal pain. He had no significant past medical or family history. The couple, both farmers and currently unemployed, had shared the same household for over 5 years. A 13C-urea breath test was positive, confirming active *H. pylori* infection. Colonoscopy with biopsy of the ileocecal region established a diagnosis of MALT lymphoma. Immunohistochemistry was positive for CD20, CD79a, and BCL2, and negative for CD10 and BCL6. The Ki-67 proliferation index was approximately 20%. CD21 staining showed preserved follicular dendritic cell networks. Staging PET/CT revealed only mild thickening of the terminal ileum/cecum without evidence of pathological lymphadenopathy or distant metastatic disease.

**Figure 2:**
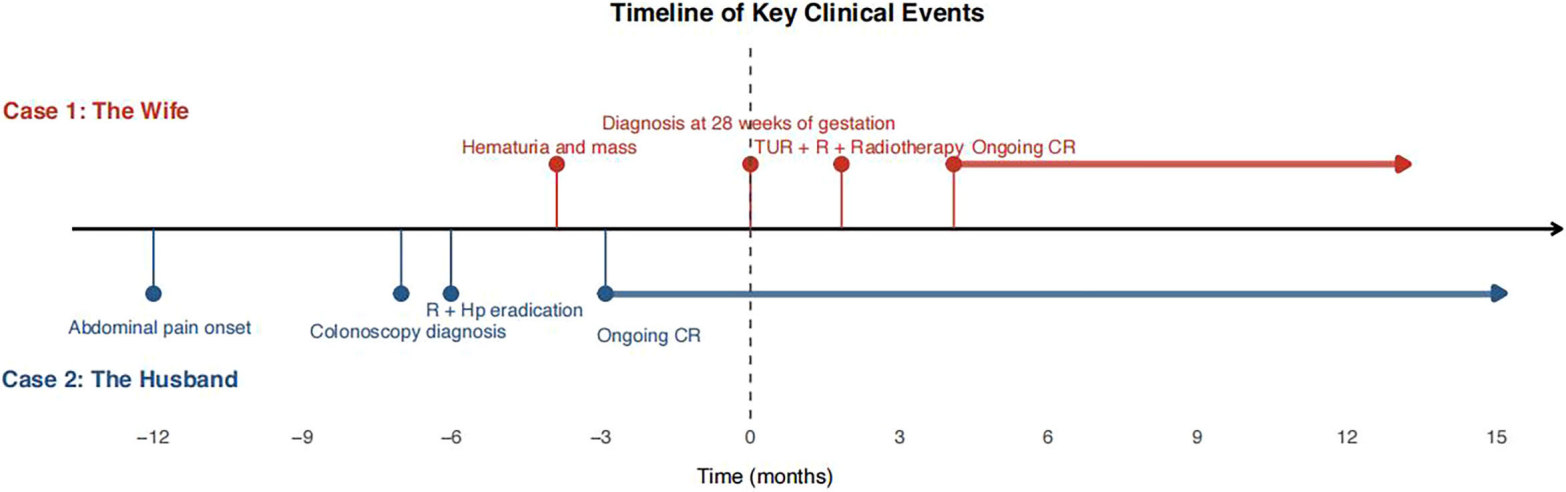
Timeline of key clinical events. The timeline illustrates the sequence of diagnosis, treatment, and follow-up milestones for the wife (upper panel) and husband (lower panel). R, rituximab; TUR, transurethral resection; CR, complete remission; Hp, Helicobacter pylori.

The patient was treated with a standard 14-day quadruple therapy for H. pylori eradication, concurrent with 4 weekly cycles of rituximab (375 mg/m² per dose). He did not receive radiotherapy. A follow-up 13C-urea breath test performed 6 months later confirmed successful eradication of H. pylori. Repeat colonoscopy at that time showed complete macroscopic resolution, with no ulceration or neoplastic lesions identified. Furthermore, at the 18-month follow-up post-treatment, the patient remained free of disease recurrence. **Figure 2** summarizes the timeline of key clinical events for both patients.

## Discussion

Extranodal marginal zone B-cell lymphoma (MZL) accounts for approximately 5–8% of all B-cell lymphomas, with MALT lymphoma being its most common subtype (4). The gastrointestinal tract, particularly the stomach, represents the predominant site of involvement, though MALT lymphoma can also arise in other locations such as the ocular adnexa, lungs, salivary glands, skin, breast, and thyroid (5). A well-established etiological link exists between *H. pylori* infection and gastric MALT lymphomagenesis. However, other infectious agents including *Chlamydia psittaci* and *Campylobacter jejuni*, as well as autoimmune disorders, have been implicated in the pathogenesis of MALT lymphoma at various anatomical sites (6, 7).

Primary lymphoma of the bladder is exceedingly rare, constituting ≤0.2% of all lymphomas. Among these, extranodal MZL is the most frequently observed histologic subtype, followed by diffuse large B-cell lymphoma. A subset of cases is associated with a history of chronic cystitis, with *Escherichia coli* being a commonly identified pathogen (8). Primary bladder MZL exhibits a female predominance, with painless gross hematuria as the most common presenting symptom, often accompanied by irritative voiding symptoms such as dysuria, nocturia, and urgency. Systemic B symptoms are typically absent. The disease follows an indolent clinical course with a low propensity for dissemination; most patients present with stage IE disease and experience prolonged survival exceeding 10 years (9). Although no standardized management guidelines exist, multiple therapeutic approaches—including transurethral resection, radiotherapy, systemic chemotherapy, immunotherapy, and antibiotic combinations—have demonstrated efficacy.

This report describes the rare occurrence of MALT lymphoma in a married couple diagnosed within a short interval. The husband presented with ileocecal MALT lymphoma and tested positive for *H. pylori*, whereas the wife was diagnosed with the even rarer entity of primary bladder MALT lymphoma and was H. pylori-negative. Both patients achieved complete remission following therapy. The central question posed by this conjugal presentation is whether it represents mere coincidence or implies a shared underlying etiology.

While coincidence cannot be definitively excluded—given that MALT lymphoma, though uncommon, has a baseline population incidence—the near-synchronous diagnosis of lymphomas at two uncommon anatomical sites, especially the wife’s bladder primary, reduces the likelihood of a purely random event. We acknowledge that without a larger case series or population-based evidence, the possibility of statistical coincidence remains. Nevertheless, shared environmental exposure emerges as the most plausible explanatory hypothesis. Prolonged cohabitation implies commonalities in diet, water source, lifestyle habits, and potential exposure to environmental carcinogens or occupational factors. Notably, infectious agents may serve as a critical link. The diverse microbiology associated with MALT lymphoma raises the possibility of an as-yet-unidentified common pathogen or a similar chronic inflammatory microenvironment triggering lymphoproliferation at distinct sites. Furthermore, a shared genetic susceptibility, such as polymorphisms in immune-response genes that heighten sensitivity to common environmental triggers, cannot be entirely ruled out. In the present case, the husband’s lymphoma involved the ileocecal region, whereas the wife’s lesion was located in the urethra. This discordance in anatomical sites suggests that shared environmental exposures may create a systemic background permissive for lymphomagenesis through immunomodulatory effects, such as the induction of regulatory T−cell responses and chronic inflammatory states. However, the specific anatomical site where the disease ultimately manifests is likely determined by local co-factors within the microenvironment, including the presence of chronic inflammation, epithelial injury, or acquired mucosa-associated lymphoid tissue at that particular location.

The literature contains scarce reports of conjugal lymphoma. Kim et al. described three couples with non-Hodgkin lymphoma who had cohabitated for over 25 years and exhibited identical or highly similar histologic subtypes, supporting the role of prolonged shared infectious or environmental exposures in lymphomagenesis (10). To our knowledge, however, no previous cases of conjugal MALT lymphoma involving discordant anatomical sites such as the intestine and urinary tract have been reported, rendering the present case unique. Unlike the cases reported by Kim et al., our couple had a relatively shorter cohabitation period of approximately 5 years and presented with divergent sites of involvement, histologic features, and H. pylori status. This heterogeneity suggests that the etiology of MALT lymphoma may be more complex than currently understood and that common environmental factors could represent one contributing element.

Our study has several important limitations. First, as a single case report, it cannot establish causality. Second, due to constraints in tissue availability, we were unable to perform in-depth molecular studies on the tumor samples from both patients—such as whole-exome sequencing to identify shared somatic mutations or metagenomic sequencing to systematically screen for common pathogen-derived nucleic acids. Such investigations would be crucial for elucidating potential common etiological mechanisms.

## Conclusion

In summary, we present the first documented case of conjugal MALT lymphoma involving disparate anatomical sites. The temporal and spatial clustering strongly suggests the potential influence of shared environmental or infectious exposures. Further molecular and epidemiological studies are warranted to explore common etiological pathways of lymphomagenesis in closely cohabitating individuals.

## Data Availability

The original contributions presented in the study are included in the article/supplementary material. Further inquiries can be directed to the corresponding author.
